# Transient Pneumonitis as a Possible Adverse Reaction to the BNT162b2 COVID-19 mRNA Vaccine in a Patient with Rheumatoid Arthritis: A Case Report and Review of the Literature

**DOI:** 10.1155/2022/3124887

**Published:** 2022-08-23

**Authors:** Yusuke Ohkubo, Shin-ichiro Ohmura, Ryuhei Ishihara, Toshiaki Miyamoto

**Affiliations:** Department of Rheumatology, Seirei Hamamatsu General Hospital, Hamamatsu, Shizuoka, Japan

## Abstract

The coronavirus disease (COVID-19) pandemic caused by severe acute respiratory syndrome coronavirus 2 has led to rapid progress in vaccine development to prevent the spread of the disease. Although COVID-19 vaccines have excellent effectiveness in reducing morbidity and disease severity with minor adverse reactions, some patients develop late hypersensitivity events as autoimmune reactions such as rheumatoid arthritis, lupus nephritis, and vasculitis following COVID-19 vaccination. Herein, we describe a case of pneumonitis following COVID-19 mRNA vaccination in a patient with rheumatoid arthritis, which resolved spontaneously.

## 1. Introduction

The development of coronavirus disease (COVID-19) vaccines has progressed rapidly. Several vaccines have been reported to be effective in reducing morbidity and disease severity [1, 2]. In Japan, the mRNA vaccines BNT162b2 (Pfizer-BioNTech) and mRNA-1273 (Moderna) have been approved. Although minor adverse reactions are common, few serious adverse reactions have been reported [1, 2]. In addition, there have been late hypersensitivity events as autoimmune reactions such as rheumatoid arthritis (RA), lupus nephritis, and vasculitis following COVID-19 vaccination [3–6]. Herein, we describe a case of pneumonitis following COVID-19 mRNA vaccine, thought to be due to an autoimmune reaction, in a patient with RA, which resolved spontaneously.

## 2. Case Presentation

**Figure 1:**
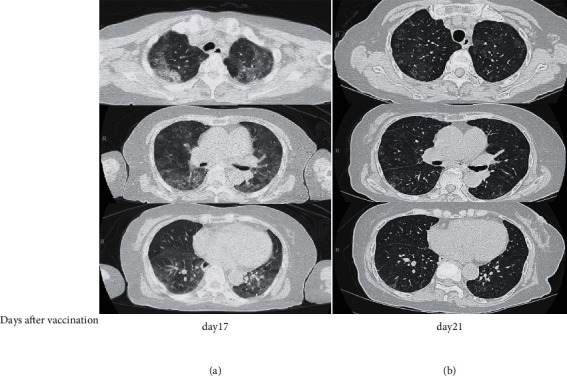
Chest computed tomography scans showing changes in the lung lesions. (a) CT scan performed on Day 17. (b) CT scan performed on Day 21. The second CT scan shows a clear improvement compared to the CT scan performed 4 days earlier. CT: computed tomography.

A 76-year-old woman with stable RA developed malaise and a high-grade fever (>39.0°C) 5 days after receiving a second dose of BNT162b2 mRNA vaccine. Ten days after vaccination, she developed a persistent cough and dyspnea. Seventeen days after vaccination, she was admitted to another hospital because of persistent fever. Blood tests showed a white blood cell (WBC) count of 4990/*μ*L and lactate dehydrogenase (LDH) and C-reactive protein (CRP) levels of 477 U/L and 6.4 mg/dL, respectively. Although the antigen test for SARS, serum B-D glucan, and blood culture was negative, tests for *Mycoplasma*, *Chlamydia*, and sputum culture were not examined. Chest computed tomography (CT) showed infiltration and ground glass opacites (GGOs) in all lung fields (Figure 1(a)). The patient was provisionally diagnosed with interstitial lung disease (ILD) and was transferred to our hospital 21 days after vaccination.

Prior to the onset of her symptoms, the patient's RA had been stable on methotrexate treatment (12 mg/week), which she had taken for 15 years.

On admission, her body temperature, blood pressure, pulse, respiratory rate, and SpO_2_ were 36.5°C, 110/60 mmHg, 65 beats/min, 18 breaths/min, and 98% (in room air), respectively. Her arthritis was stable and showed no changes of note since a previous check-up one month earlier. The results of blood tests were as follows: WBC, 5420/*μ*L; red blood cells (RBC), 377 × 10^4^/*μ*L; hemoglobin (Hb), 11.2 g/dL; hematocrit, 35.5%; platelets, 40.4 × 10^4^/*μ*L; CRP 1.47 mg/dL; LDH, 311 U/L; Krebs von den Lungen‐6 (KL-6), 452.4 U/mL; and surfactant protein D (SP-D), 226 ng/mL. Her rheumatoid factor, anti-cyclic citrullinated peptide antibody, and antinuclear antibody were negative.

Tests for *Mycoplasma*, *Chlamydia*, and *tuberculosis* (T-Spot), and antigen tests for *Cryptococcus*, *Candida*, *Aspergillus*, cytomegalovirus antigen, and SARS-CoV-2 antigen were negative. Serum *β*-D glucan, blood culture, and polymerase chain reaction testing of respiratory samples for *Pneumocystis jirovecii* DNA were also negative. Chest CT showed a significant improvement compared to the chest CT performed 4 days earlier (Figure 1(b)). The patient was treated conservatively and did not receive antibiotics or immunosuppressive drugs during hospitalization. Her cough, dyspnea, and fever resolved spontaneously, and she was discharged from hospital after 7 days. Her final diagnosis was transient pneumonitis induced by a COVID-19 mRNA vaccine. At the 3-month follow-up, she had not experienced a relapse of pneumonitis and her blood test results had improved: CRP, 0.06 mg/dL (from 1.47 mg/dL); LDH, 189 U/L (from 311 U/L); KL-6, 165.1 U/mL (from 452.4 U/mL); and SP-D, 102 ng/mL (from 226 ng/mL).

## 3. Discussion

To the best of our knowledge, this is the first report of transient pneumonitis following COVID-19 mRNA vaccination in a patient with RA. The patient's CT scan showed bilateral GGOs, which was consistent with ILD. The diagnosis of ILD in patients with RA is challenging because ILD has several causes, including bacterial pneumonia, viral pneumonia, *Pneumocystis* pneumonia, RA, and certain drugs. Drug-induced ILD is particularly difficult to diagnose because the clinical, radiological, and histological findings are non-specific and other causes of ILD must be excluded [11]. Bronchoscopy is required to diagnose drug-induced ILD. However, the Japan Society for Respiratory Endoscopy recommends that unnecessary bronchoscopy should be avoided during the COVID-19 pandemic [7]. In our patient, we did not perform bronchoscopy based on the Japan Society for Respiratory Endoscopy recommendation because blood tests did not show any sign of infection, including *Mycoplasma*, *Chlamydia*, or *Pneumocystis* infection, and her blood culture was negative. The patient had been taking the same drugs for 15 years, and the only new medication that she had received was the COVID-19 mRNA vaccine. Furthermore, her symptoms developed 5 days after the vaccination and the pneumonitis improved spontaneously with decreasing serum KL-6 and SP-D, without any relapse. These findings suggest that the COVID-19 mRNA vaccine induced transient pneumonitis. However, several investigators reported that the serum KL-6 and SP-D is associated with pulmonary diseases with alveolar inflammation, not only in ILD but also in respiratory infections, such as *Pneumocystis* pneumonia, COVID-19, and viral bronchitis [8–10, 12]. We could not exclude all respiratory diseases including infectious pneumonia because of the lack of bronchoscopic confirmation and tests for all respiratory infections with increasing KL-6 and SP-D.

The BNT162b2 mRNA vaccine contains RNA, lipids including polyethylene glycol (PEG), and a buffer, but unlike other vaccines, it contains no adjuvants or preservatives. Compared to conventional vaccines, mRNA vaccines have several advantages: they are non-infectious, do not contain cellular components, induce cellular immunity, do not require adjuvants, are simple to produce, and are inexpensive [2, 13]. The spike protein of the virus adsorbs to cell membranes covered with lipid bilayers and receptor-binding proteins on the body's immune cells, which react with these proteins to produce neutralising antibodies. PEG, one of the components of the vaccine, can cause anaphylactic reactions [14]. The overall risk of anaphylaxis following vaccination with COVID-19 mRNA vaccines is low, and almost all adverse reactions are minor [15]. However, severe adverse reactions, including ILD, have been reported following vaccination [16, 17].

We conducted a literature search for studies published up to October 2021 to determine the characteristics of patients who developed pneumonitis after the COVID-19 mRNA vaccination and found four case reports (Table 1) [16–19]. All patients had received the BNT162b2 vaccine. Of the five patients (the four previously reported cases and our patient), pneumonitis developed in two patients after the first dose and in three patients after the second dose. All patients developed the symptoms within five days after vaccination. The median age was 66 years (range, 60–86 years), and four of the five patients were male. Chest CT showed bilateral GGOs in all patients. Four patients received immunosuppressive therapy, and all patients survived. Our patient developed symptoms five days after vaccination with the BNT162b2 vaccine, which is consistent with the other cases. Notably, unlike the other cases, our patient's pneumonitis resolved spontaneously without the use of immunosuppressive therapy.

The mechanism of pneumonitis development following COVID-19 vaccination is unclear. In contrast, in patients with pneumonitis after influenza vaccination, two major mechanisms are involved: cytotoxicity of the vaccine itself and an autoimmune response to the vaccine [20]. As with patients who develop pneumonitis following COVID-19 vaccination, almost all patients with pneumonitis after influenza vaccination develop bilateral GGOs on chest CT within 10 days and recover after immunosuppressive therapy [16, 21]. The risk factors for developing pneumonitis after vaccination are unknown. According to the World Health Organization, there is no association between vaccination and the development of autoimmune diseases in patients with no underlying disease [22]. Several investigators have reported that systemic rheumatic diseases (SRD) did not worsen after influenza, pneumococcal, or human papillomavirus vaccination [23–25]. According to the 2019 European League against Rheumatic Diseases (EULAR) recommendations for vaccination in adult patients with autoimmune inflammatory rheumatic diseases, influenza vaccination is recommended for patients with RA [26]. In addition, the American College of Rheumatology and EULAR recommend COVID-19 vaccination for patients with SRD.

It is difficult to predict severe adverse reactions to COVID-19 mRNA vaccines because they are the first mRNA vaccines to be developed. Although serious adverse reactions, such as thrombosis with thrombocytopenia, myocarditis, and Guillain–Barré syndrome, have been reported after COVID-19 vaccination [27–29], risk factors for these adverse reactions have not been identified. Further prospective studies are warranted to investigate severe adverse reactions following COVID-19 vaccination, including pneumonitis.

In conclusion, COVID-19 mRNA vaccines may induce autoimmune diseases, including pneumonitis, and clinicians should be aware of the possibility of these diseases following COVID-19 vaccination.

## Figures and Tables

**Table 1:** Characteristics of patients with ILD after COVID-19 vaccination.

	Case				
	1	2	3	4	5
Age (years)	86	65	60	66	76
Sex	M	M	M	M	F
Comorbidities	HT, DM, CKD	OMI, HL, HT	ACO, HT	None	RA, HT, myoma
Smoking	Never	Ex-smoker	Ex-smoker	NS	Never
Allergy history	NS	None	None	NS	Hay fever
Vaccine type	Pfizer-BioNTech	Pfizer-BioNTech	Pfizer-BioNTech	Pfizer-BioNTech	Pfizer-BioNTech
Dose	First	First	Second	Second	Second
Time from vaccination to symptom onset (days)	1	5	4	2	5
Symptoms	Fever, cough, dyspnea	Fever, dyspnea	Dyspnea	Fever, cough, dyspnea	Fever, cough
CRP (mg/dL)	11.4	5.5	10.9	8.7	6.4
Bilateral GGO on chest CT	Yes	Yes	Yes	Yes	Yes
Immunosuppressive therapy	Yes	Yes	Yes	Yes	No
Outcome	Survived	Survived	Survived	Survived	Survived
Reference	[7]	[8]	[9]	[10]	Current case

*Note*. ACO: asthma and chronic obstructive pulmonary disease overlap; CKD: chronic kidney disease; CRP: C-reactive protein; CT: computed tomography; DM: diabetes mellitus; F: female; GGO: grand glass opacity; HL: hyperlipidemia; HT: hypertension; ILD: interstitial lung disease; M: male; NS: not specified; OMI: old myocardial infarction; RA: rheumatoid arthritis.

## Data Availability

All relevant data are included within the article.
